# “Eugenics talk” and the language of bioethics

**DOI:** 10.1136/jme.2007.021592

**Published:** 2008-05-29

**Authors:** S Wilkinson

## Abstract

In bioethical discussions of preimplantation genetic diagnosis and prenatal screening, accusations of eugenics are commonplace, as are counter-claims that talk of eugenics is misleading and unhelpful. This paper asks whether “eugenics talk”, in this context, is legitimate and useful or something to be avoided. It also looks at the extent to which this linguistic question can be answered without first answering relevant substantive moral questions. Its main conclusion is that the best and most non-partisan argument for avoiding eugenics talk is the Autonomy Argument. According to this, eugenics talk per se is not wrong, but there is something wrong with using its emotive power as a means of circumventing people’s critical–rational faculties. The Autonomy Argument does not, however, tell against eugenics talk when such language is used to shock people into critical–rational thought. These conclusions do not depend on unique features of eugenics: similar considerations apply to emotive language throughout bioethics.

… opinion ought, in every instance, to determine its verdict by the circumstances of the individual case; condemning every one, on whichever side of the argument he places himself, in whose mode of advocacy either want of candour, or malignity, bigotry or intolerance of feeling manifest themselves … and giving merited honour to every one, whatever opinion he may hold, who has calmness to see and honesty to state what his opponents and their opinions really are, exaggerating nothing to their discredit, keeping nothing back which tells, or can be supposed to tell, in their favour. This is the real morality of public discussion … (Mill).^1^

In bioethical discussions of preimplantation genetic diagnosis (PGD) and prenatal screening, accusations of eugenics are commonplace, as are counter-claims that this talk of eugenics is misleading and unhelpful.^2^^–^6 This paper asks whether “eugenics talk”, in this context, is legitimate and useful or something to be avoided. It also looks at the extent to which this linguistic question can be answered without first answering relevant substantive moral questions—for example, about the permissibility of PGD. That is, can we decide on our moral language prior to, or without committing ourselves to, a particular substantive moral view? Many of the arguments discussed here have general implications for the ways in which, throughout bioethics, issues of language and of substance inter-relate.

## DEFINING *EUGENICS*

Although the concern of this paper is with whether we should use eugenics talk, not with what such talk means, it will nonetheless be useful first to say something about how *eugenics* is defined. The term was coined in 1883 by Francis Galton.^7^ He defined it as the study of “the conditions under which men of a high type are produced” and as “the science which deals with all influences that improve the inborn qualities of a race”.^8^ Eugenics, however, is not just a field of study and, as Diane Paul notes:

… it is less often identified as a science than as a social movement or policy, as in Bertrand Russell’s definition, “the attempt to improve the biological character of a breed by deliberate methods adopted to that end”.^9^

Other, similar definitions include those found in the *Oxford English dictionary*, which defines *eugenic* as “pertaining or adapted to the production of fine offspring, *esp.* in the human race”, and in the *Routledge encyclopaedia of philosophy*, which defines *eugenics* as “the attempt to improve the human gene pool”.^10^

Most would accept that “the attempt to improve the human gene pool” is at the heart of the concept (including even who those who view such attempts as misguided or immoral). However, as Paul notes, beyond this, defining *eugenics* becomes somewhat controversial and messy:

As a historian of modern genetics, I am often asked whether human genetics represents disguised, or incipient, or possibly a new kind of eugenics. Those who pose the questions may not be certain how to define eugenics, but they are almost always convinced that it is a bad thing, one which should be prevented. Indeed, fear of a eugenics revival appears to be a principal anxiety aroused by the Human Genome Project … , in Europe as well as the United States … While almost everyone agrees that eugenics is objectionable, there is no consensus on what it actually is. Indeed, one can be opposed to eugenics, and for almost anything (p536).^9^

One way of conceptualising these definitional disagreements is to see them as disputes between people who want to narrow down the concept *eugenics* in certain ways and others who want to resist these narrowings-down. For instance, some people want to say that only authoritarian (state-coerced) eugenics is really eugenics and that (so-called) “liberal eugenics” (based on individuals’ free choices) doesn’t count as eugenics; whereas others want to make a distinction *within* eugenics between authoritarian and liberal. Similarly, some people want to say that only positive eugenics (thought of as enhancement and/or encouraging the creation of people with “better-than-normal” traits) is really eugenics and that (so-called) “negative eugenics” (avoiding the births of people with diseases or “subnormal” traits) doesn’t count as eugenics; whereas others want to make a distinction *within* eugenics between positive and negative. (Habermas^5^ (p21) apparently takes the former view, contrasting “the prevention of the birth of a severely afflicted child” with “eugenic choice”.) Finally, some people want *eugenics* to be a moral term, to have wrongness (or “wrong-makingness”) built into it, such that to call something *eugenics* is to condemn it; while some others want it to be a more neutral term that can refer non-critically to actions that are entirely innocent, or even good.

This latter point is related to (though distinct from) questions about the emotive force of the term *eugenics*. During 2005, I conducted some expert interviews with (among others) UK-based academics, campaigners and health professionals. One of my aims was to discover what key participants in debates about reproductive ethics thought about the term *eugenics*. Not surprisingly, the most enthusiastic users of the term were those campaigners who are generally critical of reproductive and selection technologies:

We use it [*eugenics*] whenever we can and we won’t be distracted or diverted into using any other word, not least because it’s not a popular word. It’s not a word that people like to hear; it’s got a lot of nasty connotations. So we’re not going to try to find a more palatable word. (Campaigner)

On the other side, those with what might be termed “pro-choice” or “pro-science” views generally avoid the word:

I almost think we should ban the term. If you just say “eugenic” nobody knows what you mean. We should say what it is about the statement or the policies that we object to, and examine that. It’s like saying “you’re a fascist!” It’s an unexamined assertion that’s used for rhetorical effect, so it just seems lazy to me. It’s not a coherent or well-specified critique. And it’s very insulting to doctors … (Academic)

I think on any occasion when the issue might be raised there would probably be a desire to avoid using [the term *eugenics*] because of its pejorative connotations. It suggests Nazis before we even start to consider the issues. (Healthcare professional)

So nearly everyone agrees that *eugenics* is hugely emotive and negative. But whereas for some this is a reason to avoid it, for others this makes the term *eugenics* a good way of getting their message across.

## REASONS TO AVOID WORDS

In general, what reasons are there for avoiding a particular term (or for not using it in certain ways)? Five main candidates seem to crop up in bioethical debates.

- Bad consequences: calling x F would have bad consequences
- Offence: calling x F would cause reasonable offence
- Falsity: x is not F, so calling x F is false
- Misleadingness (strictly, misleadingness-without-falsity): x is F, but calling it F is nonetheless misleading
- Failure to respect autonomy (strictly, failure to respect autonomy without misleading): saying that x is F is neither false nor misleading, but nonetheless encourages non-autonomous belief-formation.

In what follows, I look at each of these arguments in turn, making some observations about their structure and their general strengths and weakness and seeing how they apply to the term *eugenics*.

A key question is: are there any arguments against eugenics talk that do not rely on a particular view of the substantive moral issues under discussion (for example, that PGD is not wrongfully eugenic)? Finding arguments of this kind is important for at least two reasons. First, if arguments about terminology depend on substantive moral views, then, in contested areas such as reproductive ethics there will be no prospect of agreeing the terms in which the bioethical debate should be conducted and each side will use its own partisan concepts and terms—an unwelcome situation in which rational debate will be difficult. Perhaps such stand-offs are inescapable, but we should at least try to avoid them. Second, people who are genuinely unsure about how to answer the substantive moral questions have to decide what concepts and terms to use, and, for them, being offered nothing but competing partisan schemas is confusing and unhelpful. So it would be good if we could offer some terminological guidance for the uncommitted. What follows then is a search for a non-partisan or “neutral” argument against using eugenics talk—that is, one that does not depend on taking a particular substantive view about the ethics of PGD, prenatal diagnosis and so on. For this reason, I will spend more time on the Autonomy Argument than on the others, because it seems that this is the most likely to succeed prior to, or in the absence of, a resolution of the underlying substantive moral disputes—although even this argument, as we shall see later, is hard to disentangle from the substantive bioethical issues.

## CONSEQUENCE-BASED ARGUMENTS

There are certainly cases in which the bad consequences of saying “F” make it wrong to say “F”—for example, it may be wrong to call someone an F (even if they are one) if this will cause a riot that kills 10 000 people. So we should admit that sometimes bad consequences constitute over-riding reasons to avoid saying “F”, even if we are not consequentialists and do not think that maximising good consequences is the *only* morally relevant consideration. That being said, purely consequence-based arguments will often be of limited value when we are deciding whether or not to use terms such as *eugenics*, because the different sides in the debate frequently disagree fundamentally about what counts as a good or bad consequence. For example, those opposed to PGD may think that one good consequence of calling it *eugenics* is that people will associate it with Nazism and oppose it, while those who are in favour of PGD would view this as a bad outcome. So it looks as if we need to settle the substantive questions first in order to deploy this argument. This does not mean that the argument is unsound, but it is not what I earlier termed “non-partisan”—except in those rather rare cases where there is bipartisan agreement both about what the likely consequences will be and about whether these consequences are good or bad.

## OFFENCE

Faced with accusations of “eugenics”, who might be offended? The most obvious potential victims are (the allegedly eugenicist) doctors; another group is victims of the Nazi eugenicists, who may feel that calling PGD eugenics trivialises Nazi atrocities. The claim that such people will be offended is plausible. However, as I have argued elsewhere, there is a crucial distinction between reasonable and unreasonable offence and (with some qualifications that I will not go into here) Arguments from Offence are sound only when the offence is reasonable.^11^^–^13 The rationale for this constraint is that, without it, Arguments from Offence would oblige us to pander to people’s wacky beliefs and unduly sensitive natures. What if (for example) members of a religious cult found the words *menstruation* and *pregnant* offensive? Would doctors and nurses be obliged to stop using them in public? No, because any offence taken would be unreasonable. (Obviously, if the cultists’ offence was justified, that would be another matter, but I am assuming here that it is not.) So, for *eugenics*, the question is not so much “are people offended?”, because they are, but rather “is their offence reasonable?”

This means that the Argument from Offence has very little independent argumentative power. In order to know whether it works, we need to know whether the offence felt by (for example) doctors is reasonable. One of the key determinants of this is the question of whether they really are eugenicists. If they are, then to take offence at being called a eugenicist may well be unreasonable because, arguably at least, it is unreasonable to complain about someone giving a truthful description of you. If, on the other hand, they are not eugenicists, then the offence probably is reasonable. So the reasonableness or otherwise of the doctors’ offence boils down to the question of whether or not they are eugenicists, which is why the Argument from Offence lacks independent force. First, it suffers from a similar problem to the consequence-based argument—namely, we cannot decide the terminological question without answering one of the major substantive questions (“are they eugenicists?”). Second, it collapses into or is parasitic on a different argument, the Falsity Argument , discussed in the next section.

Before looking at the Falsity Argument, I should mention that, contrary to what was just suggested, it is possible for people to be offended reasonably by accusations of F even if they are F. The cases I have in mind are ones where *F* has negative connotations and sends out a dual message: that A is F and that being F is bad. Thus, a sex worker might take umbrage at being called a “whore”, not because this term is descriptively inaccurate, but rather because of the negative evaluation conveyed. Similarly, a fat person may object to being *called* fat when this is done in a way that suggests that there is something morally or aesthetically wrong with fatness. (These are obviously oversimplifications but will, I hope, suffice as illustrative examples.) Even these cases, however, depend on a sort of Falsity Argument. In particular, the reasonableness of any offence taken will depend largely on whether the evaluation conveyed is itself true and justified. If it is—for example, if the person’s fatness really is reprehensible—then, arguably, taking offence is not reasonable. If the evaluation conveyed is not true, however, then taking offence is reasonable, but the moral force of any Argument from Offence will rely on an underlying appeal to falsity: on the fact that the implied moral judgement is untrue.

A further complication is that moral concepts other than offence are generally in play in these situations. Thus, calling a fat person fat may still be hurtful and unkind even if the person’s fatness is reprehensible. So it does not follow from what I have said about the reasonableness and unreasonableness of offence that it is always permissible to point out people’s faults, or that there are no moral constraints on how people’s faults are discussed.

## FALSITY AND MISLEADINGNESS

The Falsity Argument against using *F* says simply that (other things being equal) we shouldn’t call x F if x is not F. So, in the case of *eugenics*, the Falsity Argument against using the term is that many of the doctors who are *called* eugenicists are not eugenicists. This would, of course, be contested by their critics, many of whom sincerely believe that they are. All I can say about this argument, then, is that it takes us straight to an impasse and is unlikely to help resolve the linguistic issues unless we already have answers to some of main the substantive questions, such as “is PGD eugenics?”

Turning to misleadingness: sometimes saying that x is F can mislead people even if x really is F. An example of this is an incident that occurred in Wales when a paediatrician was mistaken for a paedophile by some linguistically challenged vandals and returned home “to find the outside of her property daubed with the words “paedo” ”.^14^ At least in some places, then, calling someone “a paediatrician” is misleading, not to mention dangerous, even if she really is a paediatrician. Hence, other terms (such as “children’s doctor”) might be preferable.

Thinking along similar lines, some doctors might (in private) concede that they are eugenicists, in a non-pejorative technical sense of the word, but wouldn’t want to be branded “eugenicist” because the public understand *eugenics* in a narrow and negative way and may think, for example, that eugenicists are all Nazis. However, against this, some critics of reproductive technologies will assert that the public’s negative views of eugenics are quite right and hence the public would not have been misled if they came to think that all eugenicists believe in something akin to Nazism. Thus, the Misleadingness Argument, like the Falsity Argument, takes us to an impasse, since whether people are misled or not depends on the answer to one or more of the substantive ethical questions (for example, about whether eugenics must involve something akin to Nazism).

Another version of the Misleadingness Argument says that using the term *eugenics* is often misleading or confusing because (as was noted earlier) there is too much disagreement or confusion about the meaning of the term. According to this argument, given that several different definitions of eugenics are in play (*Eugenics*^def1^, *Eugenics*^def2^, *Eugenics*^def3^, *Eugenics*^def4^ and so on) and that different people will therefore take eugenics claims to mean different things, it is best to avoid the term altogether in order to avoid spreading confusion.

There seem to be two problems with this version of the Misleadingness Argument against eugenics talk. First, there are ways of reducing or eliminating such misunderstandings, notably, getting speakers to say what they mean by *eugenics*. Thus, one person may say that they are using *Eugenics*^def1^, while another admits to using *Eugenics*^def4^. The debate can then proceed with clarity and, even if participants disagree about what the best or correct definition is, they can at least know what each other mean. Obviously, this will not be practicable in all situations (in news media encounters, for example, there will rarely be an opportunity to define terms), but getting people to say what they mean could deal with many of the possible misunderstandings.

Second, there is a consistency or “absurd implications” problem with the argument. For if we *in general* stopped using terms with contested definitions or those about whose meaning there was confusion, then (at least in ethics) we wouldn’t be left with many terms. All of the following key terms (to name just a few) have contested definitions and using them can cause confusion: *autonomy*, *bioethics*, *consent*, *euthanasia*, *freedom*, *harm*, *health*, *justice* and *person*. But this is not a sufficient reason to give up on these terms (especially given the point just made about the possibility of clarification). Furthermore, given the apparent importance of the underlying concepts, the chances are that people would just concoct other (perhaps equally confusing) words for autonomy, health, personhood and the like, if they were made to stop using the existing terms—which would just make matters worse.

Now, it could be argued that that people are even more confused about *eugenics* than, say, *autonomy*, although personally I find this rather implausible and it is hard to know how this could be established: through some kind of socioscientific survey of ethical confusion, perhaps. Alternatively, one might argue that, whereas these other bioethical concepts or terms are worth saving, *eugenics* is not. But this auxiliary argument is based on considerations other than the fact that there is too much disagreement or confusion about the meaning of *eugenics* and so takes us beyond the Misleadingness Argument, rather than being a defence or a version of the argument. Furthermore, for reasons discussed earlier, I suspect that people’s views about whether the idea of the term *eugenics* is worth saving will be closely tied to their substantive moral views about the subject of eugenics; hence, we will once again arrive at a partisan impasse.

I conclude, therefore, that the Misleadingness Argument against eugenics talk is quite weak, although clearly we should all strive to avoid using the term (and indeed all other terms) in misleading ways and should, where practicable, define our terms.

## FAILURE TO RESPECT AUTONOMY

The idea here is that there are some ways of communicating that, without lying or misleading, fail to respect people’s autonomy: methods of communication that circumvent or neutralise people’s critical–rational faculties. Non-rational persuasion is, of course, ubiquitous and it would be wildly implausible to suggest that it is always wrong. However, it may be wrong in some circumstances, and it seems plausible to suppose that we usually respect people’s autonomy more when we use rational rather than non-rational persuasive means and that there should therefore be a presumption in favour of rational means. This idea underlies the Autonomy Argument against using eugenics talk.

Sometimes, when addressing bioethical issues, people use images to generate “moral intuitions”. For example, there are numerous antiabortion and antivivisection websites that contain gruesome images of aborted fetuses and mutilated animals. In such cases, there is not (or need not be) any lying or distortion taking place (that is, the alleged pictures of aborted fetuses are, as far as I can tell, really pictures of aborted fetuses), but even so, and even if abortion were wrong, there would still be a worry regarding autonomy about this way of getting the message across. One part of the concern is that the use of images bypasses people’s critical–rational faculties and constitutes manipulation^i^. 15 16 Another is that we should not encourage people to base their ethical views on the way things look, since this is an ineffective way of acquiring moral knowledge.

The Autonomy Argument against eugenics talk then focuses on the fact that the word *eugenics* is highly emotive. The problem with emotive language is that, like explicit images, it encourages people to disengage their critical–rational faculties and to form moral views based on irrelevant or superficial features. So, given that all sides agree that eugenics talk is emotive, there seems to be a strong (and non-partisan) Autonomy Argument against using it: the claim being that eugenics talk, especially if part of a concerted effort to alter others’ moral beliefs, is a failure to fully respect people’s autonomy, because it attempts to circumvent their critical–rational faculties.

## OBJECTIONS TO THE AUTONOMY ARGUMENT

The Autonomy Argument faces several objections.

The first is simply an appeal to counter-examples. Take, for instance, the terms *genocide* and *Nazi*. Surely it is okay to call genocide *genocide* and Nazis *Nazis* even though these terms are highly emotive, more so than *eugenics*. So why doesn’t the same go for *eugenics*: what’s wrong with calling eugenics *eugenics*? A lot depends on the details of the case and, even in the case of the Nazis, there may sometimes be a defensible Autonomy Argument for not *calling* them *Nazi*s. Imagine, for example, that we are having an academic–historical debate about whether an individual Nazi was responsible for a crime. One can certainly imagine circumstances in which referring to the person as “a Nazi” would cloud people’s judgements and encourage them not to use their critical–rational faculties. So these examples, *genocide* and *Nazi*, are not compelling; like *eugenics*, it may sometimes be wrong to use these terms, and for the very same reasons.

The second objection says that it is acceptable to use emotive language when this is the best way of getting people to hold true beliefs. Perhaps this is not so much an objection to the Autonomy Argument as an independent argument against its conclusion and what we have here is a clash between competing values or principles: respect for people’s autonomy versus the desirability of true belief. We cannot reasonably assert that true belief will never win out, and there probably are some extreme cases in which true belief should be prioritised over autonomy. Nonetheless, there are some (perhaps non-decisive) reasons for having a fairly strong presumption in favour of allowing autonomy to prevail.

To start with, there is a non-accidental connection (though not one I will attempt to explain here) between forming beliefs autonomously, through a process of independent deliberation and reasoning, and having true beliefs. In particular, deliberation and reasoning, at least in the long term, deliver a better truth ratio (the proportion of a person’s beliefs that are true). If this is the case, then it justifies a presumption in favour of allowing and encouraging people to form beliefs in this way, although there may be exceptional cases in which non-rational means are the best way of getting people to believe particular truths. Moreover, for one person to impose views on another using non-rational persuasion smacks of a certain kind of arrogance, of a failure to recognise one’s own epistemic shortcomings (or the possibility of such shortcomings), and perhaps also of a certain cowardice about exposing one’s own ideas to rational critique. These are the kinds of concerns that people have about religious cults and charismatic political leaders: that they aim to promulgate their beliefs non-rationally, rather than offering them to us as candidates for independent critical assessment.

Finally, a third objection to the Autonomy Argument accuses it of relying on an extreme form of linguistic Puritanism. One way of framing the problem is: can the Autonomy Argument advocate the avoidance of emotive language in bioethics without implausibly entailing its avoidance in other areas such as the arts? It looks as if the answer to this must be “no”, because emotive language will often have the very same effects in the arts as it does in bioethics: that is, it will (sometimes) bypass people’s critical–rational faculties and affect their views, by non-rational means. Thus, proponents of the Autonomy Argument must apparently be against all but the most sober artistic expression, and this looks like a reductio ad absurdum.

This is not, however, a decisive objection, although a certain amount of bullet-biting is required. The first move in defending the Autonomy Argument is to point out that emotive language can be deployed for various purposes. One is to foist beliefs on people by circumventing their critical–rational faculties. This, it seems to me, is a prima facie objectionable use of emotive language, and it can arise in the arts, bioethics, TV advertising and elsewhere. (This admission of generality is the bullet-biting.) However, there are many other purposes to which emotive language can be put. One is to cause aesthetic or emotional experiences (without manipulating people’s beliefs). Another is to *encourage* people to use their critical–rational faculties, perhaps by shocking them into thinking about something previously unquestioned. The importance of this is that when emotive language is used in the arts, it is not always, or even usually, done to foist beliefs on people. More often than not, it has other aims and so is not a target of the Autonomy Argument. Thus, while (as I have conceded) the Autonomy Argument condemns *some* artistic works for their use of emotive language (when they aim, like propaganda, at belief-manipulation), it is not as puritanical as was suggested earlier, and it is compatible with a permissive view of much artistic emotive language.

In this way, the Autonomy Argument is saved from the apparent reductio ad absurdum. However, if the arts and bioethics are essentially the same (as far as emotive language is concerned), then the defence used by artists can also be used by bioethicists. In particular, users of emotive language may argue, as one of my interviewees did:

We use it [*eugenics*] in the same way as we use the word *apartheid* to talk about the discrimination that we face because that is, from our perspective, the reality. It does shock, and it *needs* to shock people into looking at the real situation for disabled people. One of the major problems is that we’re really not seen as human beings, and therefore people’s attitudes to us need to be startled … (Campaigner)

So in bioethics, as in the arts, emotive language can be used to shock people into thinking critically about something previously unquestioned and, when it is used for this purpose, there is no autonomy-based objection to it.

The Autonomy Argument, then, is partially successful. It provides us with good reasons to avoid eugenics talk, but only where such talk is a means of circumventing people’s critical–rational faculties. Eugenics talk is often used in this way, but we must also concede that it can be used in the opposite way, to arouse and engage people’s critical–rational faculties; in these cases there is no failure to respect autonomy.

## CONCLUSIONS

It seems that the best and most non-partisan argument for avoiding eugenics talk is the Autonomy Argument. According to the version of this argument just outlined, eugenics talk per se is not wrong. However, there is something wrong with using its emotive power as a means of circumventing people’s critical–rational faculties: a process that is analogous to the use of gruesome images of fetuses and animals to persuade people to oppose abortion and animal experimentation. Such methods can bypass or distort people’s reasoning processes and, when this happens, there can be a failure fully to respect their autonomy.

However, in defence of eugenics talk, we have also seen that it may be justified when it is used to “shock people into” thinking critically about subjects that they otherwise wouldn’t—provided of course that these are subjects that they *ought to* think about or have views on (thus, I presume, shocking people into thinking about crochet or train-spotting would be unjustified). In addition, there may be cases in which over-riding autonomy is justified in order to get people to hold significant true beliefs. So even the Autonomy Argument cannot be separated entirely from substantive bioethical views, because questions about which views are important and/or true will determine the extent of its applicability.

These conclusions do not depend on unique features of eugenics, and so similar considerations apply to emotive language throughout bioethics. For instance, describing abortion as “butchering unborn children” or NHS resource limitations as “the government’s euthanasia programme”^17^ will perhaps fall foul of the Autonomy Argument; but then again, if these words are used to shock people into a critical–rational thought process, using them may be permissible or good.

## Footnotes

i Manipulation is a complicated and underexplored moral concept. For further discussion, see Kligman and Culver^15^ (pp173–97) and Rudinow^16^ (pp338–47).

## References

[1] Mill JS On liberty. London: Longman, Roberts & Green, 1869

[2] Davies A A disabled person’s perspective on eugenic abortion. London: Society for the Protection of Unborn Children (SPUC), 2003 http://www.spuc.org.uk/documents/papers/e-0079b.pdf (accessed 20 April 2008)

[3] Gillott J Screening for disability: a eugenic pursuit? J Med Ethics 2001;27 (Supp II):ii21–ii23 11574654 10.1136/jme.27.suppl_2.ii21 PMC1765546

[4] King D Preimplantation genetic diagnosis and the ‘new’ eugenics. J Med Ethics 1999;25: 176–82 10226925 10.1136/jme.25.2.176 PMC479204

[5] Habermas J The future of human nature. Cambridge: Polity, 2003

[6] Holm S Harris J Free speech, democracy, and eugenics. J Med Ethics 2004;30:519 15574434 10.1136/jme.2004.010959 PMC1733955

[7] Coutts M McCarrick P Eugenics. Kennedy Inst Ethics J 1995;5:163–78, p163 10143183

[8] Galton F Essays in eugenics. Honolulu: University Press of the Pacific, 1909:35

[9] Paul D Is human genetics disguised eugenics? Ruse M Hull D, eds. Biology and philosophy. Oxford: Oxford University Press, 1998: 536–49

[10] Chadwick R Genetics and ethics. In: Craig E, ed. Routledge encyclopedia of philosophy. London: Routledge, 1998

[11] Garrard E Wilkinson S Mind the gap: the use of empirical evidence in bioethics. In: Hayry M Takala T Herissone-Kelly P, eds. Bioethics and social reality. Amsterdam: Rodopi, 2005:73–87

[12] Sheldon S Wilkinson S Female genital mutilation and cosmetic surgery: regulating non-therapeutic body modification. Bioethics 1998;12: 263–85 11657294 10.1111/1467-8519.00117

[13] Wilkinson S Bodies for sale: ethics and exploitation in the human body trade. London: Routledge, 2003:212–3

[14] BBC News Online Paediatrician attacks ‘ignorant’ vandals, 30 August 2000. http://news.bbc.co.uk/1/hi/wales/901723.stm (accessed 20 April 2008)

[15] Kligman M Culver C An analysis of interpersonal manipulation. J Med Philos 1992;17:173–97 1588243 10.1093/jmp/17.2.173

[16] Rudinow J Manipulation. Ethics 1978;88:338–47

[17] Harris J The value of life. London: Routledge, 1985:85

